# O7-1 The association of occupational and leisure time physical activity with all-cause mortality. Using an individual participant dataset (N = 634,131)

**DOI:** 10.1093/eurpub/ckac094.049

**Published:** 2022-08-29

**Authors:** Bart Cillekens, Margo Ketels, David Hallman, Nidhi Gupta, Els Clays, Huysmans Maaike, Holtermann Andreas, Pieter Coenen

**Affiliations:** 1 Department of Public and Occupational Health, AmsterdamUMC, Amsterdam, The Netherlands; 2 Department of Public Health and Primary Care, Ghent University, Gent, Belgium; 3 Department of Occupational and Public Health Sciences, University of Gävle, Gavle, Sweden; 4 National Research Centre for the Working Environment, National Research Centre for the Working Environment, Copenhagen, Denmark

**Keywords:** Occupational physical activity, Meta analysis, Cohort studies

## Abstract

**Background:**

Physical activity is a key determinant for health and considered as an important factor in the prevention of lifestyle related-diseases. All physical activity domains are generally considered to be health enhancing. However, accumulating evidence in recent years suggests that occupational physical activity may not have the same beneficial health effect as leisure time physical activity. Our aim was to assess the association of occupational and leisure time physical activity and all-cause mortality.

**Methods:**

We obtained individual participant data from published and unpublished cohort studies and assessed their risk of bias. We harmonized the data, and used Cox survival regression models to assess the association between occupational and leisure time physical activity with all-cause mortality, in a two-stage individual participant data meta-analysis. Different models were performed to assess the impact of relevant confounders including behavioral, health-related and socio-economic factors. Results of the data were reported with hazard ratios (HRs) and 95% confidence intervals (95% CI).

**Results:**

Data from 22 prospective cohort studies showed that male workers with high occupational physical activity had an increased risk of all-cause mortality in comparison with sedentary occupational physical activity (HR: 1.12, 95%CI: 1.03- 1.23). For female workers, no such association was found (HR: 1.01, 95%CI: 0.85-1.19). when comparing high with sedentary occupational physical activity.

Increasing levels of leisure time physical activity were inversely and dose-dependently associated with a reduced risk of all-cause mortality. For example, high compared with sedentary leisure time physical activity was associated with reduced risks for males (HR: 0.53, 95%CI: 0.36-0.79) and for females (HR: 0.49, 95%CI: 0.31-0.79).

All associations remained robust when adjusting for additional relevant confounders, leaving one study out analysis, and when assessing the role of bias and reverse causality.

**Conclusion:**

We consistently found a reduced risk of all-cause mortality with increasing levels of leisure time physical activity, but not for occupational physical activity. These findings indicate that occupational activity may not be health-enhancing. These findings suggest that occupational physical activity may not be considered a suitable substitute to leisure time physical activity when striving for health enhancement.

